# Diverse tick-borne microorganisms identified in free-living ungulates in Slovakia

**DOI:** 10.1186/s13071-018-3068-1

**Published:** 2018-09-03

**Authors:** Mária Kazimírová, Zuzana Hamšíková, Eva Špitalská, Lenka Minichová, Lenka Mahríková, Radoslav Caban, Hein Sprong, Manoj Fonville, Leonhard Schnittger, Elena Kocianová

**Affiliations:** 1 Institute of Zoology, Slovak Academy of Sciences, Dúbravská cesta 9, 845 06 Bratislava, Slovakia; 2 Institute of Virology, Biomedical Research Center, Slovak Academy of Sciences, Dúbravská cesta 9, 845 05 Bratislava, Slovakia; 3 Široká 22, 831 07 Bratislava, Slovakia; 4 Laboratory for Zoonoses and Environmental Microbiology, National Institute for Public Health and Environment, 9 Antonie van Leeuwenhoeklaan, P.O. Box 1, Bilthoven, The Netherlands; 5 Instituto de Patobiologia, CICVyA, INTA-Castelar, 1686 Hurlingham, Prov. de Buenos Aires Argentina; 6 CONICET, C1033AAJ Ciudad Autónoma de Buenos Aires, Argentina

**Keywords:** Wildlife, Tick-borne pathogens, *Anaplasma phagocytophilum*, *Theileria*, Slovakia

## Abstract

**Background:**

Free-living ungulates are hosts of ixodid ticks and reservoirs of tick-borne microorganisms in central Europe and many regions around the world. Tissue samples and engorged ticks were obtained from roe deer, red deer, fallow deer, mouflon, and wild boar hunted in deciduous forests of south-western Slovakia. DNA isolated from these samples was screened for the presence of tick-borne microorganisms by PCR-based methods.

**Results:**

Ticks were found to infest all examined ungulate species. The principal infesting tick was *Ixodes ricinus,* identified on 90.4% of wildlife, and included all developmental stages. Larvae and nymphs of *Haemaphysalis concinna* were feeding on 9.6% of wildlife. Two specimens of *Dermacentor reticulatus* were also identified. Ungulates were positive for *A. phagocytophilum* and *Theileria* spp. *Anaplasma phagocytophilum* was found to infect 96.1% of cervids, 88.9% of mouflon, and 28.2% of wild boar, whereas *Theileria* spp. was detected only in cervids (94.6%). Importantly, a high rate of cervids (89%) showed mixed infections with both these microorganisms. In addition to *A. phagocytophilum* and *Theileria* spp., *Rickettsia helvetica*, *R. monacensis*, unidentified *Rickettsia* sp., *Coxiella burnetii*, “*Candidatus* Neoehrlichia mikurensis”, *Borrelia burgdorferi* (*s*.*l*.) and *Babesia venatorum* were identified in engorged *I. ricinus*. Furthermore, *A. phagocytophilum*, *Babesia* spp. and *Theileria* spp. were detected in engorged *H. concinna.* Analysis of *16S* rRNA and *groEL* gene sequences revealed the presence of five and two *A. phagocytophilum* variants, respectively, among which sequences identified in wild boar showed identity to the sequence of the causative agent of human granulocytic anaplasmosis (HGA). Phylogenetic analysis of *Theileria 18S* rRNA gene sequences amplified from cervids and engorged *I. ricinus* ticks segregated jointly with sequences of *T. capreoli* isolates into a moderately supported monophyletic clade.

**Conclusions:**

The findings indicate that free-living ungulates are reservoirs for *A. phagocytophilum* and *Theileria* spp. and engorged ixodid ticks attached to ungulates are good sentinels for the presence of agents of public and veterinary concern. Further analyses of the *A. phagocytophilum* genetic variants and *Theileria* species and their associations with vector ticks and free-living ungulates are required.

**Electronic supplementary material:**

The online version of this article (10.1186/s13071-018-3068-1) contains supplementary material, which is available to authorized users.

## Background

In the northern hemisphere, the majority of vector-borne diseases are caused by tick-borne pathogens [1–4]. *Ixodes ricinus* is a highly competent vector for a variety of disease agents in humans as well as in livestock, such as viruses, bacteria and protozoan parasites [5–7]. Also, the bites of *I. ricinus* by themselves can cause meat allergy [8]. *Ixodes ricinus* is a generalist tick that infests more than 300 different vertebrate species [9], including birds, lizards, small rodents, hares, hedgehogs as well as free-living ruminants, carnivores or wild boar. It has a three-host life-cycle with larvae feeding predominantly on small mammals or birds, nymphs feeding on small as well as large mammals, and adults preferring larger mammals [10]. *Ixodes ricinus* is usually associated with deciduous and mixed forests, but recent studies have shown that its populations can also be abundant in green periurban and urban areas [7].

Free-living ungulates are essential feeding hosts for *I. ricinus* and play a vital role in the propagation of this species [11–15]. In addition, they are reservoirs of tick-borne microorganisms some of which may cause disease in humans and domestic animals [7, 16–18]. Knowledge of the tick-borne pathogen reservoir role of wildlife is a prerequisite for a thorough understanding of the epidemiology of tick-borne zoonotic diseases and the development of effective control measures.

The epidemiology of the obligate intracellular bacterium *Anaplasma phagocytophilum,* the causative agent of tick-borne fever in ruminants and human granulocytic anaplasmosis (HGA), is very complex in Europe, with various ecotypes involved in different epidemiological cycles [19, 20]. Presence of diverse *A. phagocytophilum* genetic variants has been reported in a wide range of free-living and domestic animals [21–25]. Among them, cervids have been suggested as reservoirs for several *A. phagocytophilum* variants transmitted by *I. ricinus*. Variants associated with roe deer (*Capreolus capreolus*) are probably non-pathogenic to humans, dogs, horses or domestic ruminants, whereas red deer (*Cervus elaphus*) is likely a reservoir for variants pathogenic to domestic ruminants and horses [20, 26, 27]. The role of wild boar (*Sus scrofa*) in the transmission cycle of *A. phagocytophilum* is still unclear. Recent molecular studies have shown that *A. phagocytophilum* genetic variants infecting wild boars and humans clustered together [26, 28, 29]. However, the short duration of infection and, as compared to deer species, the relatively low number of ticks feeding on them, question wild boar as a relevant reservoir host [30].

Wildlife is a potential source of infection with piroplasmids *Babesia* spp. and/or *Theileria* spp. Zoonotic species of *Babesia*, including *B. divergens* and *B. venatorum*, are transmitted by *I. ricinus* and have been reported in European cervids [31, 32]. It should be noted, however, that reports on the occurrence of *B. divergens* previous to its exact sequence definition by Malandrin et al. [33] have to be taken with caution as this species is highly similar to *B. capreoli. Babesia divergens* causes babesiosis in cattle and immunocompromised humans [32, 34], whereas *B. capreoli*, prevalent in roe deer, is non-pathogenic in domestic ruminants [33, 35]. Besides *B. divergens, B. venatorum* (formerly *Babesia* sp. EU1) has been found to cause disease in humans [36, 37]. Its presence has been confirmed in cervids in many European countries [32, 38–42], and recently in caprines [39] and mouflon [42]. Also, the non-zoonotic *B. odocoilei*-like taxon, *Babesia* sp. MO1 and *Babesia* sp. CH1, have been detected in cervids [39, 42, 43], and *B. motasi*, transmitted by *Haemaphysalis* spp. ticks and causing disease in sheep and goat [44], has been reported in free-living caprines [39].

In Europe, asymptomatic infections caused by piroplasmids of the genus *Theileria* such as *T. capreoli* isolates *Theileria* sp. 3185/02 and *Theileria* BAB1158 and *Theileria* spp. isolate *Theileria* sp. OT3 obtained from roe deer, red deer, and chamois, and *Theileria* sp. ZS TO4 isolated from red deer have been described in several free-living cervids and caprines [42, 45–52]. Up to now, none of these *Theileria* species have been described to cause zoonotic disease [32]. Their vectors have not been confirmed in central Europe, but probably *I. ricinus* and/or *Haemaphysalis* spp. ticks are involved in their transmission [48–50].

Free-living ruminants may be involved in the epidemiology of Q fever by maintaining *Coxiella burnetii*, whereby ticks might also play a role in the circulation of the agent and its transmission from wildlife to domestic animals [53–56]. However, the role of free-living ungulates in the epidemiology of *C. burnetii* may differ between ecosystems and geographic areas [56].

In contrast, free-living ungulates are likely not reservoirs for *Rickettsia* spp. of the spotted fever group (SFG) and *Borrelia burgdorferi* (*s*.*l*.), even though the presence of DNA specific for these bacteria have been sporadically detected in their tissues [57–60]. It is assumed that due to complement-mediated killing, the presence of *B. burgdorferi* (*s*.*l*.) in ticks feeding on cervids and wild boar is reduced [61–63].

The aims of the present study were to (i) increase the knowledge on the diversity of tick-borne bacteria and piroplasmids infecting free-living ungulates, and (ii) investigate the role of free-living ungulates as carriers of infected ticks and/or reservoirs of tick-borne pathogens in Slovakia, central Europe.

## Methods

### Study area and biological samples

Tissue samples and ticks were obtained from a total of 92 gunshot game animals of five species. Forty-four cervids comprised of the three species: roe deer (*Capreolus capreolus*; *n* = 14), red deer (*Cervus elaphus*; *n* = 8) and fallow deer (*Dama dama*; *n* = 22). The remaining two species were mouflon (*Ovis musimon*; *n* = 9) and wild boar (*Sus scrofa*; *n* = 39). Animals were shot by hunters and samples were kindly provided during the legal hunting seasons of 2011–2014 in hunting districts located in deciduous forests of the Small Carpathian Mountains (southwestern Slovakia) (Additional file 1: Figure S1). The mountains are, in part, densely forested, with average annual temperatures of 7–9 °C and an annual rainfall of 650–690 mm. Sessile oak (*Quercus petraea*) and European hornbeam (*Carpinus betulus*) dominate at lower, whereas European beech (*Fagus sylvatica*) dominate at higher altitudes [64]. The highest mountain peak reaches an elevation of 768 metres above sea level (masl).

The biological samples contained spleen from all hunted specimens, blood, lower parts of legs with skin and hoofs, and engorged and/or unattached ticks. Tick larvae and nymphs were predominantly collected from lower parts of legs and hoofs of cervids. Engorged nymphs and adults attached to other body parts were collected only from three fallow deer individuals whose whole skins were available. After sampling, tissues and ticks were preserved in 70% ethanol. Information on sex and age could not be obtained for all hunted animals and was therefore not considered in the subsequent analyses. The species, developmental stage, and gender were identified for each tick under a stereomicroscope according to Siuda [65].

### DNA isolation

Genomic DNA was isolated from subsamples of spleen, blood, and from a randomly selected collection of ticks including *I. ricinus* larvae that have been sampled in pools, and *I. ricinus* nymphs and adults and *Haemaphysalis concinna* larvae and nymphs that have all been sampled individually. Whenever available, at least five specimens of each tick species and developmental stage were used to isolate genomic DNA by applying the Macherey-Nagel NucleoSpin® Tissue kit (Düren, Germany) following the instructions of the manufacturer. Quantity and quality of the isolated DNA were measured with a Nanodrop 2000c spectrophotometer and samples were stored at -20 °C for further analyses.

### PCR detection of microorganisms

Samples were screened for the presence of DNA specific for the tested microorganisms by using polymerase chain reaction (PCR)-based assays. A real-time PCR targeting a 77-bp long fragment of the *msp2* gene of *A. phagocytophilum* was performed according to Courtney et al. [66] as described in Svitálková et al. [67]. To identify *A. phagocytophilum* variants*,* two positive samples from each ungulate species and 16 randomly selected DNA samples of engorged *I. ricinus* larvae were further analysed by a nested PCR and quantitative real-time PCR (qPCR), respectively, with primers targeting a 546-bp fragment of the *16S* rRNA gene [68, 69] and a 530-bp fragment of the *groEL* gene [70]. A 99-bp fragment of the “*Candidatus* Neoehrlichia mikurensis” *groEL* gene was amplified with a qPCR [71–73]. A PCR targeting the *com1* gene encoding a 27-kDa outer membrane-associated immunoreactive protein was applied for the detection of *C. burnetii* [74]. *Rickettsia* spp. were detected by amplifying a 381-bp fragment of the *gltA* gene using genus-specific primers [75, 76]. The presence of *B. burgdorferi* (*s*.*l*.) DNA was detected by amplification of the 5S-23S (rrfA-rrlB) intergenic spacer and identification of *Borrelia* genospecies was done using a restriction fragment length polymorphism (RFLP) assay [77]. Amplification of a 450-bp region of the *18S* rRNA gene of *Babesia*/*Theileria* was carried out by PCR following the protocols of Casati et al. [78] and Hamšíková et al. [79]. A duplex qPCR targeting a 62-bp long fragment of the *18S* rRNA gene [80] and a 104-bp fragment of an internal transcribed spacer (ITS) region was performed for the detection of *Babesia* spp.

All primers and probes used in the PCR reactions and the respective references are listed in Additional file 2: Table S1.

### Sequence analysis

Amplicons derived from randomly selected samples positive for *Rickettsia* and *Babesia*/*Theileria* and those for the partial *16S* rRNA and *groEL* genes of *A. phagocytophilum* were purified and analysed by sequencing using forward and reverse PCR primers (Macrogen, Amsterdam, Netherland). Nucleotide sequences were manually edited using the MEGA6 software [81]. Determined sequences of *A. phagocytophilum*, *Babesia* spp. and *Theileria* spp. were deposited in the GenBank database (Additional file 2: Tables S2, S3).

### Phylogenetic analysis

For the phylogenetic analysis of *Babesia* parasite sequences, a multiple alignment of determined and related *18S* rRNA gene sequences available on GenBank using ClustalW was done for piroplasmids pertaining to *Babesia* (*sensu stricto*) (Clade VI as defined in [31]). The alignment length comprised of 459 bp and consisted of 39 sequences including *T. annulata* as outgroup. Gaps were eliminated to result in a final alignment of 403 positions. The evolutionary distance was estimated using the K2 + G model with G = 0.36 to generate a neighbour-joining tree [82, 83]. For the phylogenetic analysis of *Theileria* parasite sequences, a multiple alignment of determined and related *18S* rRNA gene sequences available on GenBank using ClustalW was done for piroplasmids pertaining to *Theileria* (*sensu stricto*) (Clade V as defined in [31]) infecting large ruminants. The alignment length comprised of 464 bp and consisted of 39 sequences including *T. equi* as outgroup. Gaps were eliminated to result in a final alignment of 443 positions. The K2 + G model with G = 0.66 was used to generate a neighbour-joining tree [82, 83]. For alignment and phylogenetic analysis, the MEGA6 software was used [81].

*Anaplasma phagocytophilum groEL* gene sequences were aligned using MUSCLE. For phylogenetic analysis, 55 sequences were used: 12 from this study (see Additional file 2: Table S2), 42 from different European sources available on GenBank, and *Anaplasma marginale* (GenBank: AF165812) as the outgroup. The evolutionary history was inferred by using the Maximum Likelihood method based on the Tamura 3-parameter model [84]. Positions containing gaps and missing data were eliminated, and there were 464 positions in the final dataset. Alignment and evolutionary analyses were conducted in MEGA X [85].

### Statistical analysis

Infection rates for pooled samples of *I. ricinus* larvae were analysed by the maximum likelihood estimation (MLE) method according to Biggerstaff et al. [86]. Chi-square test was used to analyse differences in *A. phagocytophilum* and *Theileria* spp. infection rates in game species, in *I. ricinus* feeding on cervids, and in tick developmental stages. Furthermore, this test was applied to evaluate differences in single and co-infection rates between developmental stages of *I. ricinus* and between ticks originating from different cervid hosts. Results on the prevalence of *A. phagocytophilum* and *Theileria* spp. in *I. ricinus* attached to cervids were used to calculate the probability of co-infections with the two microorganisms by Chi-square test. *P* < 0.05 was considered significant in all statistical analyses. Analyses were performed by using PAST Version 3.19. [87].

## Results

### Infection rates in ungulates

*Theileria* spp. were found to infect exclusively cervids, whereas *A. phagocytophilum,* besides cervids, also infected mouflon and wild boar (Table 1). Infections with other tick-borne microorganisms were not detected in any of the examined animals. Infection rates with *A. phagocytophilum* significantly differed when cervids, mouflon, and wild boar were compared (Table 1), but no significant difference was found between cervids and mouflon (*χ*^2^ = 1.090; *P* = 0.780). The total infection rates with *Theileria* spp. did not significantly differ between cervid species; however, spleen of red deer was significantly less infected than in roe deer and fallow deer (Table 1). Mixed infections with *A. phagocytophilum* and *Theileria* spp. were detected in 89.8% of cervids (average co-infection rate for all species), but the differences in co-infection rates between individual cervid species were not significant (Table 1).

**Table 1:** Molecular detection of tick-borne microorganisms in spleen and blood of free-living ungulates. Values represent numbers of positive/examined samples and infection rates (%)

	*Capreolus capreolus*	*Cervus elaphus*	*Dama dama*	*Ovis musimon*	*Sus scrofa*	*χ*^2^-value	*P-*value^a^
Total infection (spleen and/or blood)							
*A. phagocytophilum*	13/14 (92.9)	8/8 (100)	21/22 (95.4)	8/9 (88.9)	11/39 (28.2)	44.244	<0.001
*Theileria* sp.	13/14 (92.9)	8/8 (100)	20/22 (90.9)			0.767	ns
Mixed infection	11/14 (78.6)	8/8 (100)	20/22 (90.9)			2.546	ns
Spleen							
*A. phagocytophilum*	12/14 (85.7)	8/8 (100)	20/22 (90.9)	6/9 (66.7)	10/39 (25.6)	37.553	<0.001
*Theileria* sp.	12/14 (85.7)	3/8 (37.5)	18/22 (81.8)			7.403	<0.05
Mixed infection	11/14 (78.6)	3/8 (37.5)	17/22 (77.3)			5.108	ns
Blood^b^							
*A. phagocytophilum*	6/12 (50.0)	8/8 (100)	16/17 (94.1)	4/7 (57.1)	6/31 (19.3)	21.836	<0.001
*Theileria* sp.	8/12 (66.7)	8/8 (100)	15/17 (88.2)			4.384	ns
Mixed infection	6/12 (50)	8/8 (100)	14/17 (82.3)			7.281	<0.05
Spleen and blood^b^							
*A. phagocytophilum*	6/12 (50.0)	8/8 (100)	16/17 (94.1)	3/7 (42.8)	5/31 (16.1)	35.597	<0.001
*Theileria* sp.	8/12 (66.7)	3/8 (37.5)	15/17 (88.2)			6.813	<0.05
Mixed infection	4/12 (33.3)	3/8 (37.5)	11/17 (64.7)			3.279	ns

*Abbreviation*: ns, not significant

^a^Prevalence of *A. phagocytopilum* was compared between all examined ungulate species whereas the prevalence of *Theileria* sp. was compared between the three cervid species

^b^Blood was not available from all animals

### Ticks infesting ungulates and their infection with tick-borne microorganisms

In total, 2660 *I. ricinus* (2106 larvae, 413 nymphs, 118 females and 23 males), 284 *H. concinna* (241 larvae and 43 nymphs) and 2 *Dermacentor reticulatus* (1 nymph and 1 male) were collected from 42.4% (39/92) of the examined animals. All studied ungulate species harboured ticks: red deer (62.5%), roe deer (71.4%), fallow deer (77.3%), mouflon (55.5%), and wild boar (5.1%) (Table 2). Tick infestation was observed in roe deer, red deer, fallow deer and mouflon in May and from July to December. No ticks were collected in February, April, and June when only samples from wild boars were available. The majority of wild boars were tick-free, except for two individuals, one carrying an *I. ricinus* nymph and the other a *D. reticulatus* male. The highest number of ticks was found on a fallow deer shot in August 2014 in the district Rača, Bratislava and included 800 *I. ricinus* (777 larvae and 23 nymphs) and 215 *H. concinna* (182 larvae and 33 nymphs).

**Table 2:** Numbers of collected ticks and prevalence of infestation of free-living ungulates in the Small Carpathian Mountains (southwestern Slovakia) (2011–2014)

	Infested/examined (prevalence in %)	Tick species and no. of ticks
*Capreolus capreolus*	10/14 (71.4)	*Ir*: 618 L, 68 N, 3 F, 1 M
		*Hc*: 57 L, 9 N
*Cervus elaphus*	5/8 (62.5)	*Ir*: 55 L, 30 N, 8 F, 3 M
*Dama dama*	17/22 (77.3)	*Ir*: 1422 L, 313 N, 103 F, 18 M
		*Hc*: 183 L, 34 N
		*Dr*: 1 L
*Ovis musimon*	5/9 (55.5)	*Ir*: 11 L, 1 N, 4 F, 1 M
		*Hc*: 1 L
*Sus scrofa*	2/39 (5.1)	*Ir*: 1 N
		*Dr*: 1 M
Total	39/92 (42.4)	*Ir*: 2106 L, 413 N, 118 F, 23 M
		*Hc*: 241 L, 43 N
		*Dr*: 1L, 1 M

*Abbreviations*: *Ir*, *Ixodes ricinus*; *Hc*, *Haemaphysalis concinna*; *Dr*, *Dermacentor reticulatus*; L, larva; N, nymph; F, female; M, male

A selection of ticks (22.9%; 674/2946) (*I. ricinus*: 371 larvae in 74 pools, 177 nymphs, 72 females, 21 males; *H. concinna*: 19 larvae, 14 nymphs) were analysed for the presence of tick-borne microorganisms. In total, 82.8% of the samples were infected with at least one microorganism. The diversity of microorganisms in engorged ticks was higher than in the ungulate hosts (Table 3). In addition to *A. phagocytophilum* and *Theileria* spp.,* Rickettsia* spp., *C. burnetii*, *B. venatorum*, “*Ca.* N. mikurensis” and *B. burgdorferi* (*s*.*l*.) were detected in *I. ricinus* ticks. *Haemaphysalis concinna* were infected with *A. phagocytophilum*, *Babesia* spp. and *Theileria* spp.

**Table 3:** Diversity of tick-borne microorganisms in *Ixodes ricinus* and *Haemaphysalis concinna* ticks infesting free-living ungulates. Values represent numbers of positive/examined tick samples and prevalence (in %)

Tick/Host	*Capreolus capreolus*	*Cervus elaphus*	*Dama dama*	*Ovis musimon*	*Sus scrofa*
(i) *Ixodes ricinus*					
Larvae					
*Anaplasma phagocytophilum*	25/31 (27.7)^a^; 4^b^	5/6 (23.0)^a^; 3^b^	24/32 (22.0)^a^; 11^b^	5/5 (57.1)^a^; 4^b^	
*Rickettsia* sp.	14/31 (11.6)^a^		1/32 (0.6)^a^		
*Rickettsia helvetica*	1/31 (0.7)^a^	1/6 (3.1)^a^	3/32 (1.7)^a^		
*Rickettsia monacensis*			2/3 (21.2)^a^		
*Coxiella burnetii*			1/32 (0.6)^a^		
*Babesia venatorum*	3/31 (2.1)^a^				
*Theileria* sp.	19/31 (17.7)^a^; 7^b^	1/6 (3.1)^a^; 1^b^	11/32 (8.3)^a^; 8^b^		
*Borrelia valaisiana*	1/31 (0.75)^a^				
No. of animals infested with larvae	7	3	13	4	0
Nymphs					
*Anaplasma phagocytophilum*	30/59 (50.9); 5^b^	12/15 (80.0); 4^b^	62/101 (61.4); 9^b^	1/1 (100); 1^b^	
*Rickettsia* sp.	2/59 (3.4)	1/15 (6.7)			
*Rickettsia helvetica*	2/59 (3.4)		8/101 (7.9)		
*Coxiella burnetii*	4/59 (6.8)				
CNM	1/59 (1.7)				
*Theileria* sp.	17/59 (28.8); 7^b^	9/15 (60.0); 1^b^	22/101 (21.8); 10^b^		
*Borrelia valaisiana*			1/101 (1.0)		
*Borrelia afzelii*	1/59 (1.7)				
*Borrelia garinii*	1/59 (1.7)		1/101 (1.0)		
No. of animals infested with nymphs	9	4	12	1	1
Females					
*Anaplasma phagocytophilum*	2/3 (66.7); 1^b^	7/7 (100); 4^b^	54/58 (93.1); 14^b^	4/4 (100); 3^b^	
*Rickettsia helvetica*			8/58 (13.8)	1/4 (25.0)	
*Coxiella burnetii*			1/58 (1.7)	1/4 (25.0)	
CNM	2/3 (66.7)		1/58 (1.7)		
*Babesia venatorum*			1/58 (1.7)		
*Theileria* sp.	2/3 (66.7); 2^b^	3/7 (42.9); 3^b^	39/58 (67.2); 11^b^		
No. of animals infested with females	2	4	15	3	0
Males					
*Anaplasma phagocytophilum*	1/1 (100); 1^b^	2/3 (66.7); 1^b^	13/16 (81.2); 7^b^	1/1 (100); 1^b^	
*Rickettsia* sp.	1/1 (100)				
*Rickettsia helvetica*			1/16 (6.2)		
*Theileria* sp.	0/1 (0); 0	1/3 (33.3); 1^b^	8/16 (50.0); 4^b^		
*Borrelia valaisiana*			1/16 (6.2)		
No. of animals infested with males	1	1	8	1	0
(ii) *Haemaphysalis concinna*					
Larvae					
*Anaplasma phagocytophilum*	2/12 (16.7); 2^b^		2/6 (33.3); 1^b^	1/1 (100); 1^b^	
*Babesia* sp.	2/12 (16.7)			1/1 (100)	
No. of animals infested with larvae	5	0	2	1	0
Nymphs					
*Theileria* sp.	5/14 (35.7); 2^b^				
No. of animals infested with nymphs	2	0	2	0	0

*Abbreviation*: CNM, “*Candidatus* Neoehrlichia mikurensis”

^a^MLE, maximum likelihood estimation of infection prevalence

^b^Numbers of *A. phagocytophilum-* or *Theileria* sp.-positive ungulates from which infected ticks were collected

*Anaplasma phagocytophilum* was detected in all developmental stages of *I. ricinus* collected from cervids and mouflon, and in a few engorged *H. concinna* larvae from roe deer and fallow deer. Prevalence of infection in *I. ricinus* originating from different hosts varied (Table 3), but the differences were not significant for any of the tick developmental stages (ticks from mouflon were not included in the analyses). The overall prevalence in larvae was 27.7% MLE, in nymphs and adults it was 59.3% and 90.3%, respectively, but the differences between the tick stages were not significant. Larvae infected with *A. phagocytophilum* were collected only from hosts that tested positive for *A. phagocytophilum* (4 roe deer, 3 red deer, 11 fallow deer and 4 mouflon). Similarly, infected nymphs originated only from infected hosts (5 roe deer, 4 red deer, 9 fallow deer and 1 mouflon). Infected tick females fed on infected animals (1 roe deer, 4 red deer, 14 fallow deer and 3 mouflon), but were also collected from an uninfected fallow deer. All *A. phagocytophilum-*positive *H. concinna* larvae fed on infected cervids.

*Rickettsia* spp. were detected exclusively in all developmental stages of *I. ricinus* (Table 3). Total prevalence was 6.8% (MLE), 7.3% and 11.8% in larvae, nymphs and adults, respectively. *Rickettsia*-infected ticks were obtained from five roe deer, one red deer, 11 fallow deer and one mouflon. In 27 of 46 tick samples, the *Rickettsia* species could be identified either as *R. helvetica* (25 samples) or *R. monacensis* (2 samples), while from the remaining 19 samples the *Rickettsia* species was not determined to species level.

*Coxiella burnetii* was identified in four *I. ricinus* nymphs feeding on a roe deer (shot in September 2013), in a pool of larvae and one female from a fallow deer, and in one female from a mouflon (both shot in November 2013) (Table 3).

“*Candidatus* N. mikurensis” was detected in an engorged *I. ricinus* nymph and three females collected from roe deer and fallow deer (Table 3).

*Borrelia valaisiana* was detected in a pool of *I. ricinus* larvae attached to a roe deer and in a nymph and a male from fallow deer. *Borrelia afzelii* was detected in a nymph from roe deer and *Borrelia garinii* in two nymphs, one from roe and one from fallow deer (Table 3).

**Fig. 1:**
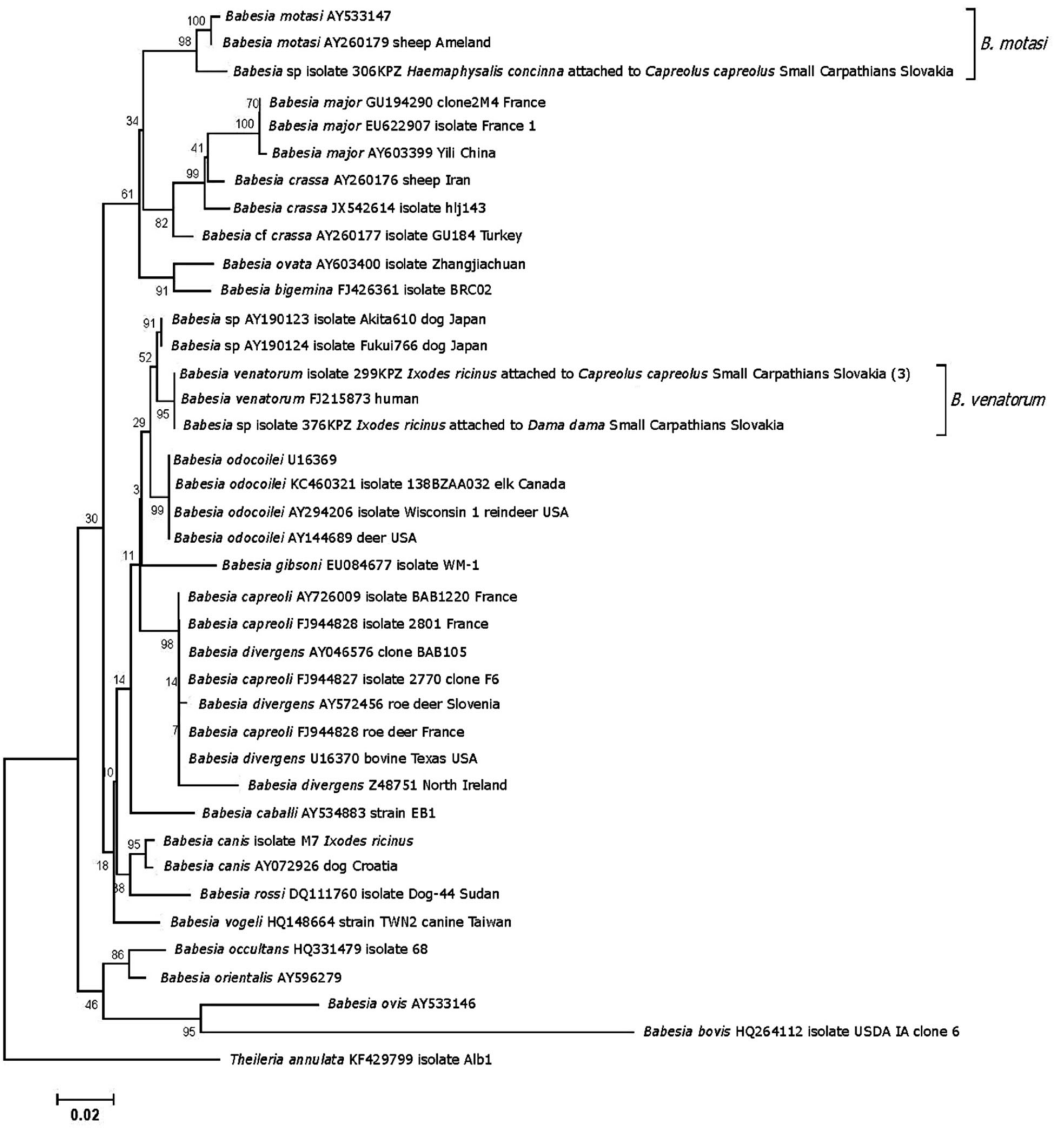
Neighbour-joining tree of hypervariable *18S* rRNA gene sequences of *Babesia* parasites. The sequence of the isolates from Slovakia is labelled with isolate designation, tick and/or vertebrate host, geographical origin, and the number of identical sequences (in parentheses). The bootstrap values based on 1000 replicates are displayed next to the branches. The tree is rooted using *Theileria annulata* as the outgroup. Clades displaying a bootstrap value of ≥ 85 are considered highly significant. The evolutionary distance is shown in the units of the number of base substitutions per site

*Babesia venatorum* (Fig. 1) was detected in three pools of *I. ricinus* larvae which each had been collected from different roe deer individuals, and in a tick female from a fallow deer. *Haemaphysalis concinna* larvae that fed on a roe deer and a mouflon each harboured another *Babesia* isolate identified as *B. motasi* (Fig. 1, Table 3).

*Theileria* spp. were present in all developmental stages of *I. ricinus* and in *H. concinna* nymphs (Table 3). Ticks that tested positive for *Theileria* spp. fed exclusively on *Theileria-*infected cervids. Total prevalence was 12.1% (MLE), 27.1%, and 58.1% in *I. ricinus* larvae, nymphs, and adults, respectively. Differences in the prevalence of infected ticks from the three cervid species were significant for larvae (*χ*^2^ = 6.731, *P* = 0.034) and nymphs (*χ*^2^ = 9.669, *P* = 0.008). Infected larvae were collected from seven roe deer, one red deer and eight fallow deer. Infected nymphs originated from seven roe deer, one red deer and ten fallow deer. Infected females were collected from two roe deer, three red deer and 11 fallow deer. *Theileria*-positive *H. concinna* nymphs fed on two infected roe deer individuals.

### Mixed infections in ticks

Mixed infections with two to four different microorganisms were found in 38.4% of *I. ricinus* samples (48.6% larval pools, 23.7% nymphs and 58.1% adults). The most common mixed infections were with *A. phagocytophilum* (31.1% larval pools, 17.5% nymphs and 52.7% adults), whereby co-infections of *A. phagocytophilum* and *Theileria* spp. prevailed (Table 4). They were detected in 16.2% larval pools, 15.2% nymphs and 38.7% adults, and occurred in ticks collected from all cervid species. Mixed infections with four microorganisms, namely *A. phagocytophilum*, *Theileria*, *Rickettsia* and *Borrelia* occurred exclusively in ticks attached to roe deer.

**Table 4:** Single and mixed infections in *Ixodes ricinus* ticks infesting free-living ungulates

Infection (Pathogens)	Tick stage	No. of samples (%)^a^	Host				
			Roe deer	Red deer	Fallow deer	Mouflon	Wild boar
Uninfected							
	L	7 pools		×	×		
	N	49	×	×	×		×
	M	3		×	×		
	Subtotal	59 (17.1)					
Single infections							
*Ap*	L	26 pools	×	×	×	×	
	N	65	×	×	×	×	
	F	23		×	×	×	
	M	8					
	Subtotal	122 (35.5)					
*Th*	L	5 pools	×	×			
	N	15	×	×	×		
	F	5	×		×		
	Subtotal	25 (7.3)					
*Rh*	N	3 (0.9)	×		×		
*Ba*	N	1 (0.3)	×				
*Bval*	N	1 (0.3)			×		
*Bgar*	N	1 (0.3)			×		
Mixed infections							
*Ap + Th*	L	12 pools	×		×		
	N	27	×	×	×		
	F	29		×	×		
	M	7		×	×		
	Subtotal	75 (21.8)					
*Ap + Bv*	L	1 pool	×				
	F	1			×		
	Subtotal	2 (0.6)					
*Ap* + *C*NM	F	1 (0.3)	×				
*Ap + Cb*	N	4	×				
	F	1				×	
	subtotal	5 (1.4)					
*Ap + R*sp	L	6 pools	×				
	N	1	×				
	M	1	×				
	Subtotal	8 (2.3)					
*Ap + Rh*	N	4			×		
	F	1				×	
	Subtotal	5 (1.4)					
*Ap + Rm*	L	1 pool (0.3)			×		
*Th + Bv*	L	1 pool (0.3)	×				
*Th + C*NM	N	1 (0.3)	×				
*Th + Rh*	L	2 pools	×		×		
	N	1			×		
	Subtotal	3 (0.9)					
*Ap + Th + C*NM	F	2	×		×		
*AP + Bv + R*sp	L	1 pool	×				
*Ap + Th + R*sp	L	6 pools	×				
	N	2	×	×			
	Subtotal	8 (2.3)					
*Ap + Th + Rh*	L	3 pools		×			
	N	1			×		
	F	8			×		
	M	1			×		
	Subtotal	13 (3.8)					
*Ap + Th + Rm*	L	1 pool (0.3)			×		
*Ap + Th + Cb*	F	1 (0.3)			×		
*Ap + Rsp + Cb*	L	1 pool (0.3)			×		
*Ap + Th + Bval*	M	1 (0.3)			×		
*Ap + Th + R*sp + *Bval*	L	1 pool (0.3)	×				
*Ap + Th + Rh + Bgar*	N	1 (0.3)	×				

*Abbreviations*: ×, presence; *Ap*, *Anaplasma phagocytophilum*; *Th*, *Theileria* spp.; *Bv, Babesia venatorum*; CNM, "*Candidatus* Neoehrlichia mikurensis"; *R*sp, *Rickettsia* sp.; *Rh*, *Rickettsia helvetica*; *Rm*, *Rickettsia monacensis*; *Ba*, *Borrelia afzelii*; *Bgar*, *Borrelia garinii*; *Bval*, *Borrelia valaisiana*; *Cb,* *Coxiella burnetii*; L, larva; N, nymph; F, female; M, male

^a^Infection prevalence (in %), the total number of analysed samples was 344 (74 pools of larvae, 177 nymphs, 72 females, 21 males)

Proportions of *I. ricinus* infected with *A. phagocytophilum* alone and co-infected with *Theileria* spp. depended on the tick stage. Significant differences were revealed between nymphs and adults (*χ*^2^ = 14.759, *P* < 0.001), but not between larvae and nymphs or adults. Proportions of single and mixed infections with *A. phagocytophilum* depended on the cervid host in larvae and nymphs, but not in adults (larval pools: *χ*^2^ = 7.755, *P* = 0.021; nymphs: *χ*^2^ = 6.127; *P* = 0.045). Overall proportions of uninfected *I. ricinus* and those infected with single and multiple pathogens did not depend on the cervid host (*χ*^2^ = 5.568, *P* = 0.234), but depended on the tick developmental stage (*χ*^2^ = 47.321, *P* < 0.001). The proportions differed significantly between larvae and nymphs (*χ*^2^ = 18.698, *P* < 0.001) and between nymphs and adults (*χ*^2^ = 40.468, *P* < 0.001), but not between larvae and adults (*χ*^2^ = 3.456, *P* = 0.178).

In *H. concinna*, mixed infection with *A. phagocytophilum* and *Babesia* sp. was detected in a single larva collected from an *A. phagocytophilum-*infected mouflon.

### *Anaplasma phagocytophilum* variants

By analysing the variation of the *16S* rRNA gene of *A. phagocytophilum* from ungulates, five variants showed identity with corresponding sequences deposited in the GenBank database. Four of these variants were designated according to Schorn et al. [88] and Silaghi et al. [89] as “Y”, “S”, “W”, and “B” (Table 5). Importantly, variant “B” from wild boar was found to be identical with the sequence of the HGA agent (AY886761). A fifth sequence obtained from mouflon (MF061301) was not identical with any of the abovementioned variants and was designated as variant “Q”. It showed 100% identity with database entries of *A. phagocytophilum* isolated from the spleen of sika deer (KU705189), red deer (KU705138), and mouflon (KU705120) in Germany as well as from blood and spleen of red deer in the Czech Republic (EU839849) and in Slovenia (AF481852), respectively. Out of 16 selected engorged *I. ricinus* larvae, amplification of *16S* rRNA gene was successful for eight ticks feeding on roe and fallow deer. Only in a single case, the *16S* rRNA gene sequence variant “S” identified in a larva corresponded with that of its fallow deer host, whereas in another larva of the same fallow deer, the variant “W” was identified. In three larvae sampled from three other fallow deer, the variant “S” was identified in one and the variant “B” in the remaining two larvae. The variant “X” was found in three larvae from roe deer.

**Table 5:** *16S* rRNA and *groEL* gene sequence variants of *A. phagocytophilum* in free-living ungulates and engorged ticks

Species/ Isolate code	Sex / Age	Variant *16S* rRNA^a^	Variant *groEl*^b^
*Capreolus capreolus* / 18SPZ^c^	♂ /?	Y	II
*Capreolus capreolus* / 55SPZ	♂ / 1 year	Y	II
*Cervus elaphus* / 19SPZ	♂ / 3 years	S	I
*Cervus elaphus* / 21SPZ	♂ / juvenile	W	I
*Dama dama* / 25SPZ	♀ / 2 years	S	I
*Dama dama* / 51SPZ	♀ / juvenile	S	I
*Ovis musimon* / 10SPZ	♂ /?	Q^e^	I
*Ovis musimon* / 63SPZ	♂ / 3 years	W	I
*Sus scrofa* / 13SPZ	♂ /?	B	I
*Sus scrofa* / 43SPZ	♀ /?	B	I
*I. ricinus* larva from *C. capreolus* / 190KPZ^d^		X	na
*I. ricinus* larva from *C. capreolus* / 193KPZ		X	na
*I. ricinus* larva from *C. capreolus* / 236KPZ		X	na
*I. ricinus* larva from *D. dama* / 129KPZ		S	I
*I. ricinus* larva from *D. dama* / 158KPZ		W	I
*I. ricinus* larva from *D. dama* / 180KPZ		S	na
*I. ricinus* larva from *D. dama* / 268KPZ		B	na
*I. ricinus* larva from *D. dama* / 382KPZ		B	na

^a^Nomenclature according to Schorn et al. [88] and Silaghi et al. [89]

^b^Nomenclature according to Jahfari et al. [20]

^c^SPZ, spleen from game

^d^KPZ, ticks from game

^e^The sequence did not match with the variants described in Schorn et al. [88] or Silaghi et al. [89] and was submitted to GenBank (accession number MF061301); na, not amplified

Analysis of *A. phagocytophilum groEL* gene sequences derived from this study (see Additional file 2: Table S2) revealed the presence of two *groEL* gene variants in ungulates that are designated as ecotype I and II according to the classification by Jahfari et al. [20]. The variant identified in wild boar showed 100% identity to the HGA agent from human blood from Slovenia (AF033101). Amplification of the partial *groEL* gene was only successful for two *I. ricinus* larvae from fallow deer (MG773209 and MG773210) that were not identical with the sequence identified in the host. In the phylogenetic tree constructed by using the 12 obtained sequences and the 42 *groEL* partial gene sequences retrieved from GenBank, the two sequences from roe deer formed a cluster together with sequences from roe deer from Germany, France, Slovenia and questing *I. ricinus* ticks from Slovenia and eastern Slovakia. The sequences from the other ungulates and engorged *I. ricinus* ticks clustered together with sequences from other sources, including that from a human patient (Additional file 3: Figure S2).

### Analysis of piroplasmid sequences

A phylogenetic tree inferred from aligned *Babesia 18S* rRNA gene sequences showed the identity of a *Babesia* isolate identified in a roe deer-attached *H. concinna* tick with *B. motasi,* with the highly significant bootstrap support of 98 (Fig. 1). Four other sequences, three amplified from *I. ricinus* ticks that had been attached to roe deer and one amplified from an *I. ricinus* attached to a fallow deer, were placed with the highly significant bootstrap support of 95 into the *B. venatorum* clade. Importantly, all four sequences were identical with the sequence FJ215873 of *B. venatorum* isolated from a human patient that has been originally isolated and described as *Babesia* sp. EU1 [36].

To assess the species identity of *Theileria* isolates, the *Theileria 18S* rRNA gene sequences were compared with corresponding sequences available on GenBank by phylogenetic analysis. In the inferred tree, *Theileria* sp. 1 and 2 sequences segregated jointly with *Theileria capreoli* isolated from red deer in Spain, and two other *Theileria* spp. isolated from red and roe deer, respectively, into a single clade. Interestingly, *Theileria* sp. 1 isolates were only found in red deer whereas *Theileria* sp. 2 isolates were only present in roe deer suggesting that these are two *Theileria* species with different host specificity (Additional file 4: Figure S3). Analysed *18S* rRNA gene sequences of *Theileria* and *Babesia* species are listed with their GenBank accession numbers in Additional file 1: Table S3.

## Discussion

Changes in land use and urbanisation increase the frequency of encounters between wildlife and domestic animals and humans that, in turn, increase the risk of contracting zoonotic diseases [18, 90–92]. Slovakia is covered, in part, by forests [64] with abundant populations of wildlife, including large game animals [93]. The present study explored associations of free-living ungulates with ticks and tick-borne microorganisms in deciduous forests of the Small Carpathian Mountains, comprising recreational areas (Bratislava Forest Park) and hunting districts.

### Infestation of ungulates with ticks

Free-living ungulates are important for maintenance of tick populations [11, 94] and serve as a reservoir and/or spillover hosts for tick-borne microorganisms [32, 95]. With respect to their wide home range, they can transport ticks of all developmental stages over long distances and thus contribute to the natural maintenance of transmission cycles of tick-borne agents and their dispersal [11, 16, 96, 97]. *Ixodes ricinus* dominated among ticks collected from the hunted ungulates in our study, which is in line with the occurrence and abundance of questing ticks in the study area [98]. Contrary to the expected, exclusively larvae and nymphs but no adults of *H. concinna* were found, though similarly as for *I. ricinus*, small mammals and ground-dwelling passerine birds are considered as the main hosts of their subadult stages and ungulates are hosts of adults [99, 100]. Tick numbers, the ratio between the abundance of developmental stages and data on seasonality of tick infestation were biased in our study because in the majority of cases only small portions of the skin restricted to a few body parts were provided and could be examined. In addition to legs and hoofs, whole skins were available only from a few fallow deer individuals from which we were able to gather relatively high numbers of ticks compared to the other game species. The majority of engorged and semi-engorged adult and subadult ticks found on the whole skin of fallow deer were attached to groins, axillae, and the belly, which is in contrast to the attachment patterns of tick developmental stages reported for roe and red deer [13, 15]. Nevertheless, our results support previous findings on the role of cervids and mouflon as hosts and vehicles for *I. ricinus* [13, 15, 58, 63, 94, 96] and the individual variation of tick burdens [11]. In contrast to reports from the Netherlands [63], but in line with findings from Poland [58], the infestation rate of wild boars was very low in our study (5.1% - only a single crawling *I. ricinus* nymph and one *D. reticulatus* male were found on their legs). We assume that the epidermis in this part of the wild boar’s body is too hard and the fur too thick to provide places favourable for tick attachment. Moreover, wild boars have been reported to be mainly hosts of *Dermacentor marginatus* [101], a species that has not been recorded in the study area [98], whereas *D. reticulatus* could be found only sporadically [67, personal observations].

### *Anaplasma phagocytopilum* in ungulates and engorged ticks

All examined game species were infected with *A. phagocytophilum.* The infection rates were high: 96.1% in cervids; 88.9% in mouflon; and 28.2% in wild boar. The presence of this bacterium in game has frequently been reported from different regions of Europe [19, 95], with varying prevalences depending on the host species, examined tissue, site, but also on the sensitivity of the detection method used. The infection rates of 100% in red deer, 95.4% in fallow deer, and 92.9% in roe deer estimated from our study are higher than values previously reported from Slovakia: red deer 17.5–53.1% [24, 57, 102]; fallow deer 66.7% [24], and roe deer 50–77% [24, 57, 102, 103]. The infection rates determined in this study are in the upper range of those confirmed by PCR in cervids from other countries of mainland Europe, where the values in red deer ranged between 1.5–86% [89, 104–114], in fallow deer between 1.5–72.5% [42, 105, 106, 108, 110, 112, 115, 116] and in roe deer between 9.6–98.9% [38, 42, 89, 105–113, 117–120]. The 88.9% infection rate in mouflon is higher than the values previously reported from Slovakia [57] and other European countries, where the prevalences ranged from 4% to 74.4% [42, 106, 110, 111]. The lower infection rate in wild boar compared to that estimated in cervids and mouflon supports former findings from Slovakia (0–16.7%) [24, 57, 112, 121] as well as from other sites in central and western Europe (0–14.3%) [28, 58, 105, 106, 110, 122–127]. However, it is necessary to note that, in addition to geographical location and habitat, the reported variations of prevalence could be due to the application of molecular detection methods of different sensitivity.

*Ixodes ricinus* is considered to represent the common vector of *A. phagocytophilum* in Europe [8] and was the most numerous tick species collected from cervids in our study, with an average prevalence of 27.2% MLE in larvae, 58.3% in nymphs and 94.3% in adults. The bacterial DNA was previously detected in questing *I. ricinus* nymphs and adults from the Small Carpathian forests [67]. The high prevalence in both cervids and attached *I. ricinus* ticks suggests that cervids may serve as reservoir hosts of *A. phagocytophilum* and are a source of infection for vector ticks in the studied region. However, the number of studies in which engorged *I. ricinus* from cervids were examined for infection with *A. phagocytophilum* along with their hosts is limited. For example, no *A. phagocytophilum* was detected in *I. ricinus* collected from roe deer in Slovakia and Poland [112, 118], but in other sites in Poland, 12.5% and 9% of ticks from roe deer and red deer, respectively, were found to be infected [58]. For engorged ticks from cervids in Italy, a 31.2% prevalence [113] or 29.9% positive pools [128] were found, but no information on the tick developmental stage was given. In another study from Italy, a prevalence of 11% and 5.4% was detected for nymphs/adults and larvae, respectively [129]. The prevalence in adult ticks feeding on cervids in Italy (7.3%) [116] and Poland (22.7%) [108] was lower than in our study, whereas it was comparable with the 86.1% prevalence reported from a site in Germany, where the infection rate of roe deer was as high as 98.7% [38]. *Anaplasma phagocytophilum* was also detected in engorged *H. concinna* larvae and nymphs feeding on infected roe deer and fallow deer. However, questing *H. concinna* from the study area were not found to be infected [67] suggesting that this species is not a competent vector of the bacterium and that the bacterial DNA originated from the ingested host blood.

Based on specific genetic markers, the presence of a wide variety of *A. phagocytophilum* variants associated with particular groups of hosts was found to circulate in wildlife and ticks in Europe [19, 95, 130, 131]. Cervids are suggested to be the main reservoir hosts, whereby roe deer probably maintain specific strains that are not pathogenic for humans or domestic livestock while red deer could be reservoirs for strains associated with disease in domestic ruminants [26, 104, 106]. According to recent findings, however, roe deer can be co-infected with two to three distinct genetic variants, including those associated with domestic ruminants [132]. Human pathogenic *A. phagocytophilum* strains have been detected in wild boars suggesting their potential reservoir role for the HGA agent [28, 122, 123]. Four ecotypes of *A. phagocytophilum* that differ in host ranges and zoonotic potential have been identified based on *groEL* gene sequences [20], whereby ecotype I is associated with the broadest host range and *I. ricinus* ticks and also includes strains causing disease in domestic animals and humans. Ecotype II was found to be associated with roe deer and does not include zoonotic strains. Sequence analysis of selected ungulate isolates from our study revealed five different variants of the partial *16S* rRNA gene and two *groEL* gene variants, whereby the results agree with previously published findings. Identification of the *16S* rRNA gene variant “B” (the prototype variant of the HGA agent) and of *groEL* sequences that are 100% identical with the HGA agent isolated from human blood from Slovenia (AF033101) in wild boars suggests that they could be potential reservoirs of the HGA strain in the study area. To our knowledge, this is the first confirmed occurrence of this strain in wild boars from Slovakia, whereas sequences of former GenBank isolates from Slovak wild boars were identical with sequences from wild ruminants, horses, dogs or wild boar and showed a lower degree of identity with the HGA sequence [24, 121]. However, further research is required to find out if wild boars in Slovakia are involved in the enzootic cycle of *A. phagocytophilum* variants pathogenic to humans. In roe deer, *16S* rRNA variant “Y” [88, 89] and *groEL* ecotype II sequences [20] were identified, which have been detected mainly in this species and have not been associated with clinical cases of granulocytic anaplasmosis [20, 38, 42, 89]. Sequences from red deer, fallow deer, and mouflon (*16S* rRNA variants “S” and “W”, *groEL* ecotype I) showed a high degree of identity with sequences from wild ruminants, cattle, horses, hedgehogs, dogs, or foxes, i.e. variants that can cause disease in domestic animals.

Four *16S* rRNA gene variants were identified in engorged *I. ricinus* larvae: variant “X”, associated with roe deer [88, 89] in larvae from roe deer, and variants “S”, “W” and “B” as well as *groEL* ecotype I in larvae from fallow deer. Interestingly, but in agreement with results for roe deer from Germany [38], not all *16S* rRNA gene variants from engorged ticks matched the variant detected in the corresponding hosts. The reasons for this variation, however, remain unclear. Nevertheless, our results support the role of cervids as natural reservoirs for several *A. phagocytophilum* genetic variants in Slovakia out of which some may be of veterinary importance.

### Piroplasmids in ungulates and engorged ticks

Previous studies have suggested the reservoir role of European cervids and caprines for different *Babesia* species, including the zoonotic *B. venatorum* and *B. divergens* [38–40, 42, 104, 111, 114, 119, 133, 134]. Infestation of cervids with ticks that carry potentially zoonotic strains of *Babesia* spp. is common [38, 58, 129, 135–137]. In the study area, infection with babesiae has previously been confirmed in questing *I. ricinus* (*B. venatorum*, *B. capreoli* and *B. odocoilei*), for which vector competence was confirmed for *B. venatorum* [138], and *H. concinna*, infected with *Babesia* spp. infective for small ruminants [79]. In the present study, the examined ungulates were *Babesia*-negative, whereas *B. venatorum* was identified in engorged *I. ricinus* larvae attached to roe and fallow deer and *B. motasi* in *H. concinna* larvae attached to roe deer. Considering the possibility of transovarial transmission of babesiae*,* our results suggest that natural foci of different *Babesia* spp., including zoonotic strains, may be present in the studied region, but further research is required to elucidate their associations with reservoir hosts. The finding of the *B. motasi* species in *H. concinna* is of particular interest and supports recent results and theories on the wide distribution of piroplasmids transmitted by this tick in Europe and Asia [31, 79, 139], and the possible role of migratory birds in their spread [140].

In contrast to the absence of *Babesia* infections, a relatively high infection rate with *Theileria* was determined in the examined cervids, corroborating findings from southwestern Hungary, where exclusively *Theileria* spp.; however, no *Babesia* spp. were identified in large game animals [141]. The presence of piroplasmids of the genus *Theileria* has been reported in wildlife from different regions of mainland Europe [40, 42, 47–51, 58, 134, 141], including a record from red deer from Slovakia dating back to 1958 [45]. *Theileria* spp. have not been associated with zoonotic infections, but chronic asymptomatic theileriosis has been observed in European cervids that may serve as infection reservoirs, with prevalences ranging up to 100% in some populations [49, 50, 134, 141]. To the best of our knowledge, this study provides the first molecular evidence of *Theileria* in cervids and the first report of *Theileria* infections in roe and fallow deer from Slovakia. *Theileria* spp. were also detected in engorged *I. ricinus* and *H. concinna* ticks feeding on infected animals, corroborating findings from other regions of Europe with abundant populations of *I. ricinus* and occurrence of *H. concinna* [49, 50]. In a phylogenetic analysis, two *18S* rRNA gene sequence variants obtained from cervids and engorged ticks clustered together with sequences designated as *Theileria* sp. and/or *T. capreoli*. This cluster included *Theileria* sp. 3185/02 from roe deer, Spain [47] and *Theileria* sp. BAB1158, Spain. Also, these variants showed an identity of 99–100% to *Theileria* sp. ZS T04 detected in red deer in Poland [48], Austria [50] and Germany [42], and also to *Theileria* spp. identified in questing *H. concinna* from Hungary [139] and Slovakia [79]. Importantly, the two genotypes *Theileria* sp. 1 and 2 can be distinguished based on a single characteristic mutation that corresponds to those recently reported in *Theileria* genotypes elaphy CE1 (exclusively identified in red and fallow deer) and capreoli CE1 (exclusively identified in roe deer) from Hungary, respectively [141]. These data strongly support the finding of Hornok et al. [141] that *Theileria* spp. of cervids comprises a complex of at least two or even more species.

The modes of transmission and vectors of *Theileria* spp. associated with European cervids are largely unknown and may depend on the abundance and dominance of tick species and the population density of cervids in a particular area [141]. Either *I. ricinus* [47–49] or *H. concinna* [50, 141] have been suggested as possible vectors, but alternative mechanisms, e.g. transplacental transmission, should also be taken into account, especially in cervid populations with high infection rates [48]. The following findings may indicate that *H. concinna* could be the vector of *Theileria* in the study area: (i) cervids are hosts for both *I. ricinus* and *H. concinna* ticks; (ii) the high *Theileria* infection rate in cervids; (iii) the detection of *Theileria* only in *I. ricinus* feeding on cervids, but not in questing ticks [79]; (vi) detection of identical *Theileria* genotypes in cervids, questing *H. concinna*, *H. concinna* attached to cervids and even in a rodent-attached *H. concinna* female [79]*.* Nevertheless, alternative mechanisms of transmission may also exist. Thus, a more in-depth molecular analysis of the detected *Theileria* genotypes and their associations with cervid hosts and vectors is needed.

### The occurrence of other bacteria in engorged ticks

In this study, SFG rickettsiae (*R. helvetica* and *R. monacensis*), *Coxiella burnetii*, “*Ca.* N. mikurensis” and *B. burgdorferi* (*s*.*l*.) were detected only in engorged *I. ricinus* ticks. Sporadic infections with *R. helvetica,* previously reported from roe deer from Slovakia [57] or roe deer and wild boar from the Netherlands [59], were not confirmed for the study area. However, our results are consistent with other studies reporting the presence of SFG rickettsiae in *I. ricinus* feeding on free-living ungulates [38, 128, 129, 142]. Moreover, rickettsial infection in tick larvae feeding on uninfected hosts as well as a comparable prevalence of SFG rickettsiae in the examined engorged *I. ricinus* and in questing ticks from the study area [143] suggest that large ungulates play a role in the dispersion of infected ticks, but are not involved in the natural circulation of SFG rickettsiae in the Small Carpathian forests.

*Coxiella burnetii* infections have been detected, e.g. in red deer [114], but not in wild boar from central Italy [127], and in red deer from the Iberian Peninsula [56], suggesting a reservoir role of red deer. In eastern Slovakia, exposure to *C. burnetii* was previously determined in game animals (up to 28.3% fallow deer and 29.6% mouflon) by routine seroscreening [144, 145]. However, there is no evidence of the involvement of large ungulates in the life-cycle of the pathogen in this country [146, 147]. Natural infections with *C. burnetii* have been detected in ticks (questing or feeding on different hosts) from different countries [53, 148] including Slovakia [74, 147, 149, 150], whereas transmission of the Q fever agent to humans *via* a tick bite is rare [53]. Based on our detections of *C. burnetii* in all developmental stages of *I. ricinus* that were collected from large ungulates we assume that similarly as for SFG rickettsiae, ungulates serve only as vehicles for the transport of infected ticks. The same seems to apply to “*Ca.* N. mikurensis” and *B. burgdorferi* (*s*.*l*.) that were previously detected in questing ticks from the study area [73, 151] and in a low portion of the examined engorged *I. ricinus,* but not in ungulates. This is in line with previous findings for “*Ca.* N. mikurensis”, for which rodents are suggested as reservoirs [152]. Free-living ungulates and *I. ricinus* feeding on them have previously been found infected with *B. burgdorferi* (*s*.*l*.) to a very low extent and are considered to be incompetent reservoirs for the pathogen. At high densities, they may even cause a decline in the density of infected ticks [63, 153].

### Mixed infections in ungulates and ticks

In our study, mixed infections with *A. phagocytophilum* and *Theileria* were found in cervids and prevailed in engorged *I. ricinus*, whereas co-infections with two to four different microorganisms were detected only in the ticks. The only mixed infection in *H. concinna* was with *A. phagocytopilum* and *Babesia* sp. Infections of free-living ungulates and their ticks with multiple microorganisms are common in natural habitats of Europe and have been reported, e.g. from Poland [58, 119], Germany [38, 42], Austria [111], Italy [114, 127, 128] and Switzerland [39]. Double infections with *A. phagocytophilum* and *Theileria* were found, e.g. in cervids from western Poland [58], but the reported co-infection rates (12.3% in roe deer and 28% in red deer) were lower than in our study.

In general, infections of ticks with multiple pathogens can result in co-transmission of various combinations of tick-borne microorganisms to vertebrate hosts, which may have severe implications for human and animal health [154–156]. We assume that co-transmission of the detected microorganisms to ungulates by ticks does not occur in the study area, or it may occur to a very low extent (e.g. co-transmission of *B. venatorum* and *A. phagocytopilum* by *I. ricinus*). This assumption is supported by the following findings: (i) ungulates were found infected only with *A. phagocytophilum* and *Theileria* and are probably reservoirs of these microorganisms; (ii) *I. ricinus* is the competent vector for *A. phagocytophilum* and *B. venatorum*, but probably not for *Theileria*; (iii) ungulates, particularly cervids, may be reservoirs of *B. venatorum*, whereas for the other recorded microorganisms their reservoir competence is questionable (SFG rickettsiae, *C. burnetii*) or has not been confirmed (*B. burgdorferi* and “*Ca.* N. mikurensis”); (iv) *H. concinna* is probably the vector of *Theileria* and *B. motasi,* but its vector competence for *A. phagocytophilum* has not been confirmed*.* On the other hand, ungulates seem to play an essential role in the dispersal of ticks that carry and may transmit or co-transmit microorganisms with possible impact on the health of domestic animals and pets [i.e. *A. phagocytophilum*, *Babesia* spp., *C. burnetii* and *B. burgdorferi* (*s*.*l*.)], and humans [i.e. the HGA agent, *B. venatorum*, “*Ca.* N. mikurensis”, SFG rickettsiae, *C. burnetii* and *B. burgdorferi* (*s*.*l*.)]. However, further research is necessary to more profoundly characterise the genotypes of the microorganisms that circulate in the study area and their pathogenicity to humans, domestic animals and pets. Positive interactions (mutualism or interactions with host symbionts), as well as competition between microorganisms co-infecting the vector ticks and the vertebrate hosts, need to be explored. Such studies have the potential to reveal (i) decreases or increases of disease severity, (ii) an increased host susceptibility to other infections, and (iii) the resulting complications for diagnosis and treatment.

## Conclusions

The results of the study demonstrate that *A. phagocytophilum* and *Theileria* circulate in natural foci of the Small Carpathian Mountains and free-living ungulates are probably their reservoirs. Also, free-living ungulates seem to be involved in the transport and dispersal of ticks infected with several microorganisms including zoonotic tick-borne pathogens, which points to the risk of exposure of hunters and tourists to multiple infections. Further studies are necessary to gain a better knowledge of the epidemiology of the tick-borne microorganisms occurring in the deciduous forests of southwestern Slovakia.

## Additional files

Additional file 1: **Figure S1.** Map of the study area. Hunting districts were located in the Small Carpathian Mountains, between Bratislava and Dubová pri Modre (see red line in the lower panel). (PDF 659 kb)

Additional file 2: **Table S1.** Primers and probes (P) used in PCR reactions and references (numbers in bold) where specific conditions of the PCR reactions are described. **Table S2.** GenBank accession numbers of *A. phagocytophilum 16S* rRNA and *groEL* gene sequences identified in spleen and engorged ticks from free-living ungulates. **Table S3.** GenBank accession numbers of piroplasmid *18S* rRNA gene sequences identified in the spleen of free-living ungulates and engorged ticks. (PDF 275 kb)

Additional file 3: **Figure S2.** Molecular phylogenetic analysis of the partial *groEL* gene of *Anaplasma phagocytophilum* derived from free-ranging ungulates and engorged *Ixodes ricinus* larvae from southwestern Slovakia. (PDF 62 kb)

Additional file 4: **Figure S3.** Neighbour-joining tree of hypervariable *18S* rRNA gene sequences of *Theileria* parasites using neighbour-joining. (PDF 332 kb)
