# Supplementary material for: Diverse tick-borne microorganisms identified in free-living ungulates in Slovakia
Source: Parasit Vectors. 2018 Sep 3;11:495. doi: 10.1186/s13071-018-3068-1 (PMC6122462; doi:10.1186/s13071-018-3068-1)
Supplement: Supplementary file 1 — Figure S1. Map of the study area. Hunting districts were located in the Small Carpathian Mountains, between Bratislava and Dubová pri Modre (see red line in the lower panel). (PDF 659 kb) [file 13071_2018_3068_MOESM1_ESM.pdf]

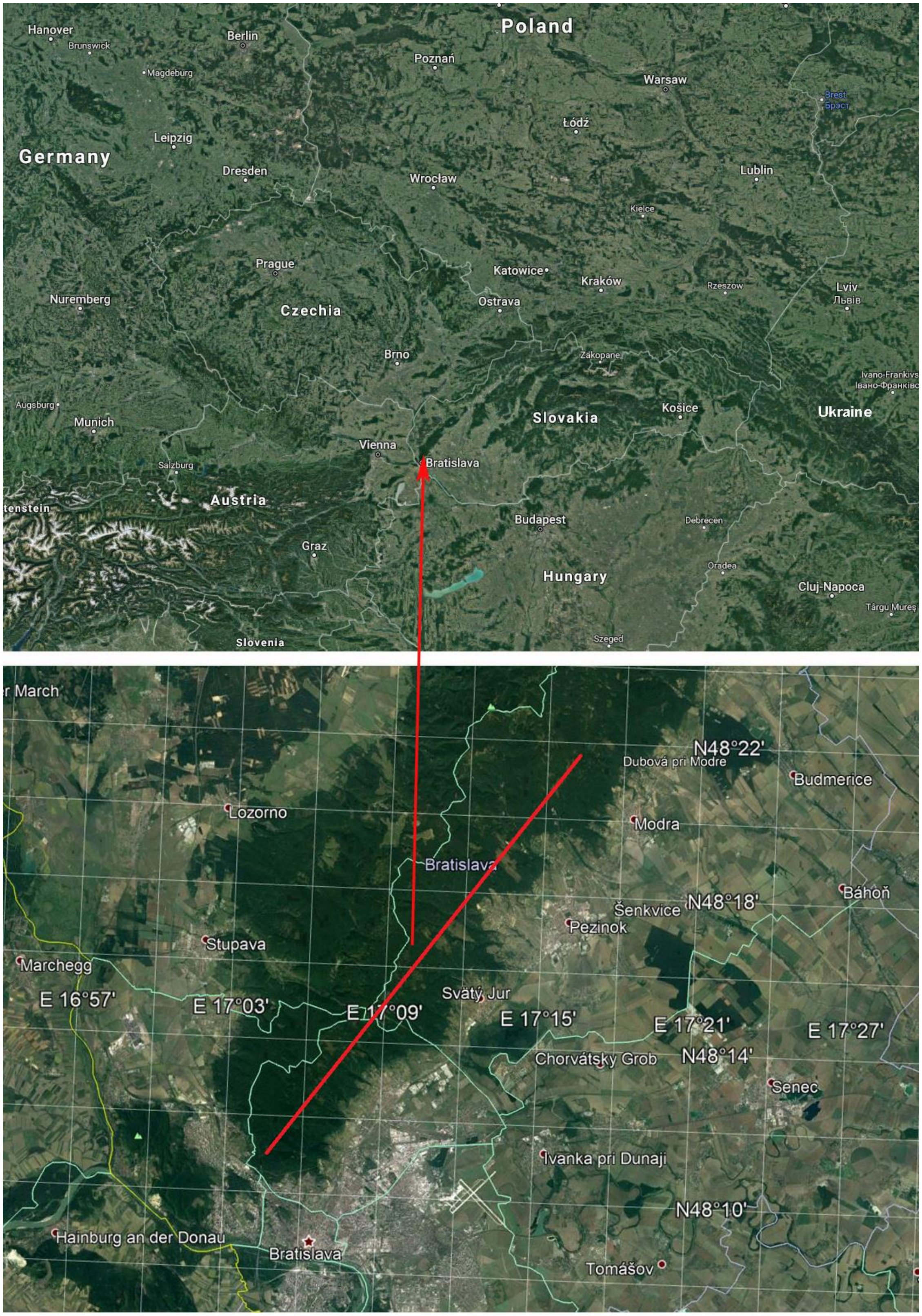

Figure S1 Map of the study area. Hunting districts were located in the Small Carpathian Mountains, between Bratislava and Dubová pri Modre (see red line in the lower panel).
