# Supplementary material for: Diverse tick-borne microorganisms identified in free-living ungulates in Slovakia
Source: Parasit Vectors. 2018 Sep 3;11:495. doi: 10.1186/s13071-018-3068-1 (PMC6122462; doi:10.1186/s13071-018-3068-1)
Supplement: Supplementary file 2 — Table S1. Primers and probes (P) used in PCR reactions and references (numbers in bold) where specific conditions of the PCR reactions are described. Table S2. GenBank accession numbers of A. phagocytophilum 16S rRNA and groEL gene sequences identified in spleen and engorged ticks from free-living ungulates. Table S3. GenBank accession numbers of piroplasmid 18S rRNA gene sequences identified in the spleen of free-living ungulates and engorged ticks. (PDF 275 kb) [file 13071_2018_3068_MOESM2_ESM.pdf]

Table S1 Primers and probes (P) used in PCR reactions and references [numbers in bold] where specific conditions of the PCR reactions are described

Organism<br>Gene	Primers and probes (P)	Sequences (5´-3´)	Refs.
A. phagocytophilum<br>*msp2*	ApMSP2f<br>ApMSP2r<br>ApMSP2p (P)	ATGGAAGGTAGTGTTGGTTATGGTATT<br>TTGGTCTTGAAGCGCTCGTA<br>HEX-TGGTGCCAGGGTTGAGCTTGAGATTG-TAMRA	[1, **2**]
A. phagocytophilum<br>16S rRNA	1st amplification:<br>ge3a<br>ge10r<br>2nd amplification:<br>ge9f<br>ge2	 CACATGCAAGTCGAACGGATTATTC<br>TTCCGTTAAGAAGGATCTAATCTCC<br><br>AACGGATTATTCTTTATAGCTTGCT<br>GGCAGTATTAAAAGCAGCTCCAGG	[3, **4**]
A. phagocytophilum<br>*groEL*	EphplgroEL-A.phago-F<br>EphgroEL-A.phago-R	ATGGTATGCAGTTTGATCGC<br>TTGAGTACAGCAACACCACCGGAA	[5, **6**] [a]
Cand. N. mikurensis<br>*groEL*	NMikGroEL-F2<br>NMikGroEL rev1<br>NMikGroEL rev2<br>NMikGroEL-P2a (P)	CCTTGAAAATATAGCAAGATCAGGTAG<br>CCACCACGTAACTTATTTAGCACTAAAG<br>CCACCACGTAACTTATTTAGTACTAAAG<br>FAM-CCTCTACTAATTATTGCTGAAGATGTAGAAGGTGAAGC-BHQ1	[7, 8, **9**]
C. burnetii<br>*com1*	CBCOS<br>CBCOE	GCTGTTTCTGCCGAACGTAT<br>AGACAACGCGGAGGTTTTTA	[**10**]
B. burgdorferi s.l.<br>5S-23S (rrfA-rrlB)<br>intergenic spacer	IgsA<br>IgsB	CGACCTTCTTCGCCTTAAAGC<br>AGCTCTTATTCGCTGATGGTA	[**11**]
Rickettsia sp.<br>*gltA*	RPCS877<br>RPCS1258	GGGGACCTGCTCACGGCGG<br>ATTGCAAAAAGTACAGTGAACA	[12, **13**]
Babesia sp./*Theileria* sp.<br>18S rRNA	BJ1<br>BN2	GTCTTGTAATTGGAATGATGG<br>TAGTTTATGGTTAGGACTACG	[14, **15**]
18S rRNA (qPCR)	Bab_18SrRNA-F<br>Bab_18SrRNA-R<br>Bab_18SrRNA-P (P)	CAGCTTGACGGTAGGGTATTGG<br>TCGAACCCTAATTCCCCGTTA<br>6-FAM CGAGGCAGCAACGG-MGB	[16] [b]
ITS (qPCR)	Bmicr_ITS_F<br>Bmicr_ITS_R<br>Bmicr_ITS_px1 (P)	CTCACACAACGATGAAGGACGCA<br>AACAGAGGCAGTGTGTACAATACATTCAGA<br>HEX-GCA +GAATTTAG+CAAAT+CAACAGG-TAMRA	This study [c]

MGB, minor groove binder group; +, Locked Nucleic Acid; BHQ, Black Hole Quencer.

If not specified otherwise, DNA lysates from questing *I. ricinus* ticks in which presence of the tested microorganisms was confirmed by PCR and sequencing served as positive controls.

[a] The positive control for *A. phagocytophilum* (q)PCRs is a DNA lysate of a spleen from a roe deer positive for *A. phagocytophilum* [6].

[b] The positive control for the *Babesia* sp. (q)PCRs is a DNA lysate of a spleen from a captive reindeer positive for *Babesia capreoli* [17].

[c] The positive control for the *B. microti* (q)PCR is a DNA lysate of a spleen from a rodent positive for *Babesia microti* (unpublished).

The multiplex qPCR for the simultaneous detection of *Cand.* N. mikurensis and *A. phagocytophilum* as well as the qPCR for *Babesia* sp. were performed using the 2x IQ-powermix (Bio-rad, Veenendaal, Netherlands) under the following conditions: 5 min 95°C, then 60 cycles of 5 s 94°C, 35 s 60°C and ending by 20 s 37°C. Detailed protocols for the qPCR are available upon request.

References to Table S1.

[1] Courtney JW, Kostelnik LM, Zeidner NS, Massung RF. Multiplex real-time PCR for detection of *Anaplasma phagocytophilum* and *Borrelia burgdorferi*. J Clin Microbiol. 2004;42:3164-8.

[2] Svitálková Z, Haruštiaková D, Mahríková L, Berthová L, Slovák M, Kocianová E, Kazimírová M. *Anaplasma phagocytophilum* prevalence in ticks and rodents in an urban and natural habitat in South-Western Slovakia. Parasit Vectors. 2015;8:276.

[3] Massung RF, Slater K, Owens JH, Nicholson WL, Mather TN, Solberg VB, Olson JG. Nested PCR assay for detection of granulocytic ehrlichiae. J Clin Microbiol. 1998;36:1090-5.

[4] Overzier E, Pfister K, Thiel C, Herb I, Mahling M, Silaghi C. *Anaplasma phagocytophilum* in questing *Ixodes ricinus* ticks: Comparison of prevalences and partial 16S rRNA gene variants in urban, pasture, and natural habitats. Appl Environ Microbiol. 2013;79:1730-4.

[5] Alberti A, Zobba R, Chessa B, Addis MF, Sparagano O, Pinna Parpaglia ML, et al. Equine and canine *Anaplasma phagocytophilum* strains isolated on the island of Sardinia (Italy) are phylogenetically related to pathogenic strains from the United States. Appl Environ Microbiol. 2005;71:6418-22.

[6] Jahfari S, Coipan C, Fonville M, van Leeuwen AD, Hengeveld P, Heylen D, Heyman P, van Maanen C, Butler CM, Földvári G, Szekeres S, van Duijvendijk G, Tack W, Rijks JM, van der Giessen J, Takken W, van Wieren SE, Takumi K, Sprong H. Circulation of four *Anaplasma phagocytophilum* ecotypes in Europe. Parasit Vectors. 2014;7:365.

[7] Jahfari S, Fonville M, Hengeveld P, Reusken C, Scholte EJ, Takken W, et al. Prevalence of *Neoehrlichia mikurensis* in ticks and rodents from north-west Europe. Parasit Vectors. 2012;5:74.

[8] Silaghi C, Woll D, Mahling M, Pfister K, Pfeffer M. *Candidatus* Neoehrlichia mikurensis in rodents in an area with sympatric existence of the hard ticks *Ixodes ricinus* and *Dermacentor reticulatus,* Germany. Parasit Vectors. 2012;5:285.

[9] Hamšíková Svitálková Z, Haruštiaková D, Mahríková L, Mojšová M, Berthová L, Slovák M, et al. *Candidatus* Neoehrlichia mikurensis in ticks and rodents from urban and natural habitats of South-Western Slovakia. Parasit Vectors. 2016;9:2.

[10] Špitalská E, Kocianová E. Detection of *Coxiella burnetii* in ticks collected in Slovakia and Hungary. Eur J Epidemiol. 2003;18:263-6.

[11] Derdáková M, Beati L, Pet'ko B, Stanko M, Fish D. Genetic variability within *Borrelia burgdorferi* sensu lato genospecies established by PCR-single-strand conformation polymorphism analysis of the rrfA-rrlB intergenic spacer in *Ixodes ricinus* ticks from the Czech Republic. Appl Environ Microbiol. 2003;69:509-16.

[12] Regnery RL, Spruill CL, Plikaytis BD. Genotypic identification of rickettsiae and estimation of intraspecies sequence divergence for portions of two rickettsial genes. J Bacteriol. 1991;173:1576-89.

[13] Špitalská E, Stanko M, Mošanský L, Kraljik J, Miklisová D, Mahríková L, et al. Seasonal analysis of Rickettsia species in ticks in and agricultural site of Slovakia. Exp Appl Acarol. 2016;68:315–24.

[14] Casati S, Sager H, Gern L, Piffaretti JC. Presence of potentially pathogenic *Babesia* sp. for human in *Ixodes ricinus* in Switzerland. Ann Agric Environ Med. 2006;13:65-70.

[15] Hamšíková Z, Kazimírová M, Haruštiaková D, Mahríková L, Slovák M, Berthová L, et al. *Babesia* spp. in ticks and wildlife in different habitat types of Slovakia. Parasit Vectors. 2016;9:292.

[16] Øines Ø, Radzijevskaja J, Paulauskas A, Rosef O. Prevalence and diversity of *Babesia* spp. in questing *Ixodes ricinus* ticks from Norway. Parasit Vectors. 2012;5:156.

[17] Bos JH, Klip FC, Sprong H, Broens EM, Kik MJL. Clinical outbreak of babesiosis caused by *Babesia capreoli* in captive reindeer (*Rangifer tarandus tarandus*) in the Netherlands. Ticks Tick Borne Dis. 2017;8:799-801.

Table S2 GenBank accession numbers of *A. phagocytophilum* 16S rRNA and *groEL* gene sequences identified in spleen and engorged ticks from free-living ungulates

Name of the isolate	Source	bp	GenBank accession number	
			16S rRNA	*groEl*
18SPZ[a]	*Capreolus capreolus* spleen	530		MF061229
55SPZ	*Capreolus capreolus* spleen	530		MF061230
19SPZ	*Cervus elaphus* spleen	530		MF061231
21SPZ	*Cervus elaphus* spleen	530		MF061232
51SPZ	*Dama dama* spleen	530		MF061233
25SPZ	*Dama dama* spleen	530		MF061234
10SPZ	*Ovis musimon* spleen	530		MF061235
10SPZ	*Ovis musimon* spleen	497	MF061301	
63SPZ	*Ovis musimon* spleen	530		MF061236
13SPZ	*Sus scrofa* spleen	530		MF061237
43SPZ	*Sus scrofa* spleen	530		MF061238
129KPZ[b]	*Ixodes ricinus* larva from *D. dama*	530		MG773209
158KPZ	*I. ricinus* larva from *D. dama*	530		MG773210

[a] SPZ, spleen from game; [b] KPZ, ticks from game

Table S3 GenBank accession numbers of piroplasmid 18S rRNA gene sequences identified in spleen of free-living ungulates and engorged ticks

Name of the isolate	Source (number of analysed samples with identical sequences)	Species	bp	GenBank accession number
1SPZ[a]	*Capreolus capreolus* spleen (11)	*Theileria* sp. 1	467	KX470610
52SPZ	*Cervus elaphus* spleen (3)	*Theileria* sp. 2	467	KX470611
87SPZ	*Dama dama* spleen (18)	*Theileria* sp. 2	467	KX470612
21KPZ[b]	*Ixodes ricinus* larva attached to *C. capreolus* (5)	*Theileria* sp. 1	467	KX470613
188KPZ	*I. ricinus* nymph attached to *C. capreolus* (6)	*Theileria* sp. 1	467	KX470614
1KPZ	*I. ricinus* female attached to *C. capreolus* (2)	*Theileria* sp. 1	467	KX470615
312KPZ	*Haemaphysalis concinna* nymph attached to *C. capreolus* (3)	*Theileria* sp. 1	467	KX470616
48KPZ	*I. ricinus* larva attached to *C. elaphus* (1)	*Theileria* sp. 2	467	KX470617
45KPZ	*I. ricinus* nymph attached to *C. elaphus* (1)	*Theileria* sp. 2	467	KX470618
59KPZ	*I. ricinus* female attached to *C. elaphus* (3)	*Theileria* sp. 2	467	KX470619
132KPZ	*I. ricinus* larva attached to *D. dama* (9)	*Theileria* sp. 2	467	KX470620
71KPZ	*I. ricinus* nymph attached to *D. dama* (9)	*Theileria* sp. 2	467	KX470621
390KPZ	*I. ricinus* female attached to *D. dama* (12)	*Theileria* sp. 2	467	KX470622
139KPZ	*I. ricinus* male attached to *D. dama* (2)	*Theileria* sp. 2	467	KX470623
299KPZ	*I. ricinus* larva attached to *C. capreolus* (3)	*Babesia venatorum*	447	KX470624
376KPZ	*I. ricinus* female attached to *D. dama* (1)	*Babesia venatorum*	447	KX470625
306KPZ	*H. concinna* larva attached to *C. capreolus* (2)	*Babesia* sp.	432	KX470626

[a] SPZ, spleen from game; [b] KPZ, ticks from game
