# Supplementary material for: Diverse tick-borne microorganisms identified in free-living ungulates in Slovakia
Source: Parasit Vectors. 2018 Sep 3;11:495. doi: 10.1186/s13071-018-3068-1 (PMC6122462; doi:10.1186/s13071-018-3068-1)
Supplement: Supplementary file 3 — Figure S2. Molecular phylogenetic analysis of the partial groEL gene of Anaplasma phagocytophilum derived from free-ranging ungulates and engorged Ixodes ricinus larvae from southwestern Slovakia. (PDF 62 kb) [file 13071_2018_3068_MOESM3_ESM.pdf]

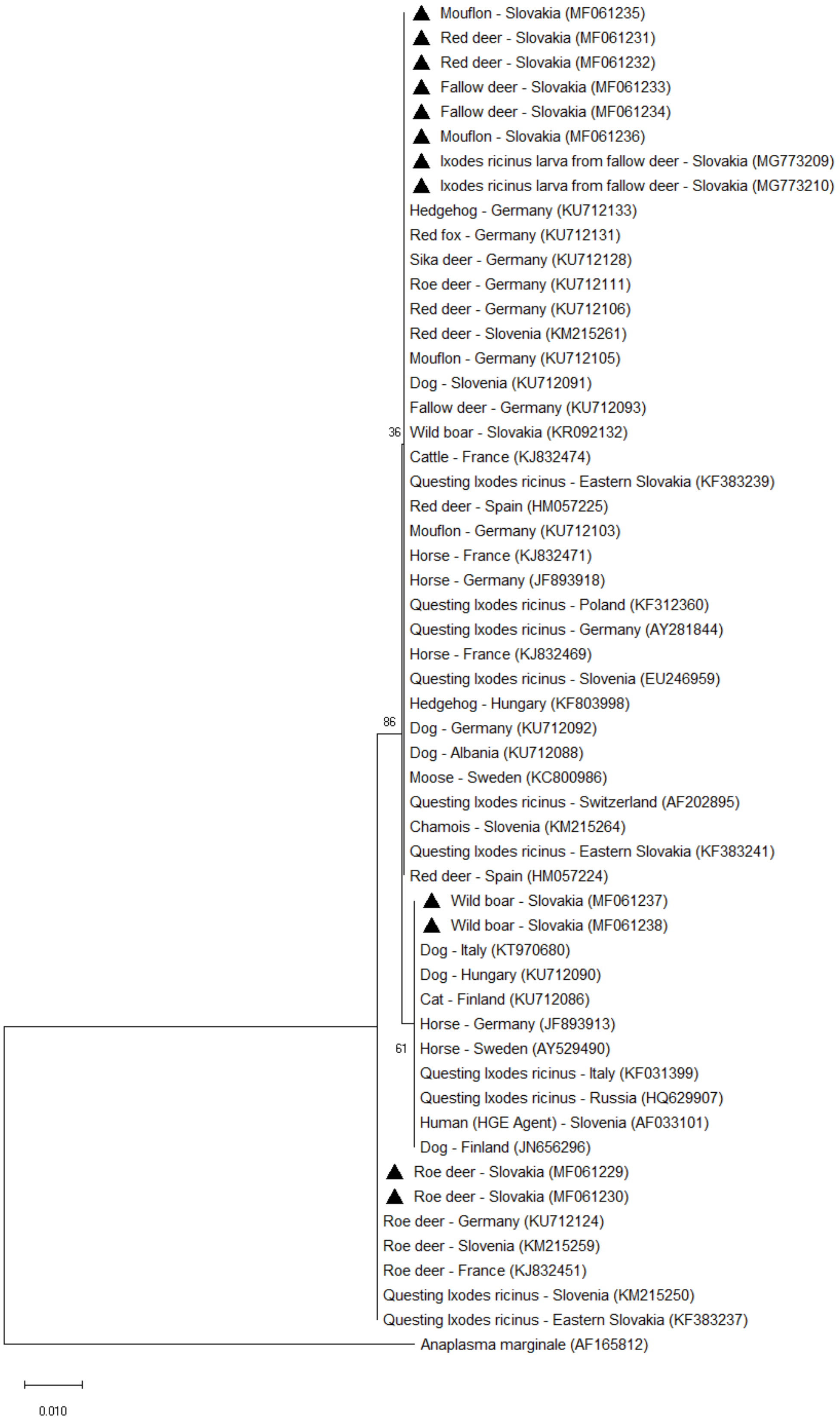
Mouflon - Slovakia (MF061235)
Red deer - Slovakia (MF061231)
Red deer - Slovakia (MF061232)
Fallow deer - Slovakia (MF061233)
Fallow deer - Slovakia (MF061234)
Mouflon - Slovakia (MF061236)
Ixodes ricinus larva from fallow deer - Slovakia (MG773209)
Ixodes ricinus larva from fallow deer - Slovakia (MG773210)
Hedgehog - Germany (KU712133)
Red fox - Germany (KU712131)
Sika deer - Germany (KU712128)
Roe deer - Germany (KU712111)
Red deer - Germany (KU712106)
Red deer - Slovenia (KM215261)
Mouflon - Germany (KU712105)
Dog - Slovenia (KU712091)
Fallow deer - Germany (KU712093)
36
Wild boar - Slovakia (KR092132)
Cattle - France (KJ832474)
Questing Ixodes ricinus - Eastern Slovakia (KF383239)
Red deer - Spain (HM057225)
Mouflon - Germany (KU712103)
Horse - France (KJ832471)
Horse - Germany (JF893918)
Questing Ixodes ricinus - Poland (KF312360)
Questing Ixodes ricinus - Germany (AY281844)
Horse - France (KJ832469)
Questing Ixodes ricinus - Slovenia (EU246959)
Hedgehog - Hungary (KF803998)
86
Dog - Germany (KU712092)
Dog - Albania (KU712088)
Moose - Sweden (KC800986)
Questing Ixodes ricinus - Switzerland (AF202895)
Chamois - Slovenia (KM215264)
Questing Ixodes ricinus - Eastern Slovakia (KF383241)
Red deer - Spain (HM057224)
Wild boar - Slovakia (MF061237)
Wild boar - Slovakia (MF061238)
Dog - Italy (KT970680)
Dog - Hungary (KU712090)
Cat - Finland (KU712086)
Horse - Germany (JF893913)
61
Horse - Sweden (AY529490)
Questing Ixodes ricinus - Italy (KF031399)
Questing Ixodes ricinus - Russia (HQ629907)
Human (HGE Agent) - Slovenia (AF033101)
Dog - Finland (JN656296)
Roe deer - Slovakia (MF061229)
Roe deer - Slovakia (MF061230)
Roe deer - Germany (KU712124)
Roe deer - Slovenia (KM215259)
Roe deer - France (KJ832451)
Questing Ixodes ricinus - Slovenia (KM215250)
Questing Ixodes ricinus - Eastern Slovakia (KF383237)
Anaplasma marginale (AF165812)
0.010

Figure S2 Molecular phylogenetic analysis of the partial *groEL* gene of *Anaplasma phagocytophilum* derived from free-ranging ungulates and engorged *Ixodes ricinus* larvae from southwestern Slovakia.

The evolutionary history was inferred by using the Maximum Likelihood method based on the Tamura 3-parameter model [84]. The tree with the highest log likelihood (-848.89) is shown. The percentage of trees in which the associated taxa clustered together is shown next to the branches. The analysis involved 55 nucleotide sequences. All positions containing gaps and missing data were eliminated. There were a total of 464 positions in the final dataset. Evolutionary analyses were conducted in MEGA X [85]. GenBank accession numbers are indicated in parentheses. Sequences from this study (see Additional file 2: Table S2) are labelled with triangles.
Initial tree(s) for the heuristic search were obtained automatically by applying Neighbor-Join and BioNJ algorithms to a matrix of pairwise distances estimated using the Maximum Composite Likelihood (MCL) approach, and then selecting the topology with superior log likelihood value. A discrete Gamma distribution was used to model evolutionary rate differences among sites (5 categories (+*G*, parameter = 200.0000)). The tree is drawn to scale, with branch lengths measured in the number of substitutions per site.
