# Supplementary material for: Diverse tick-borne microorganisms identified in free-living ungulates in Slovakia
Source: Parasit Vectors. 2018 Sep 3;11:495. doi: 10.1186/s13071-018-3068-1 (PMC6122462; doi:10.1186/s13071-018-3068-1)
Supplement: Supplementary file 4 — Figure S3. Neighbour-joining tree of hypervariable 18S rRNA gene sequences of Theileria parasites using neighbour-joining. (PDF 332 kb) [file 13071_2018_3068_MOESM4_ESM.pdf]

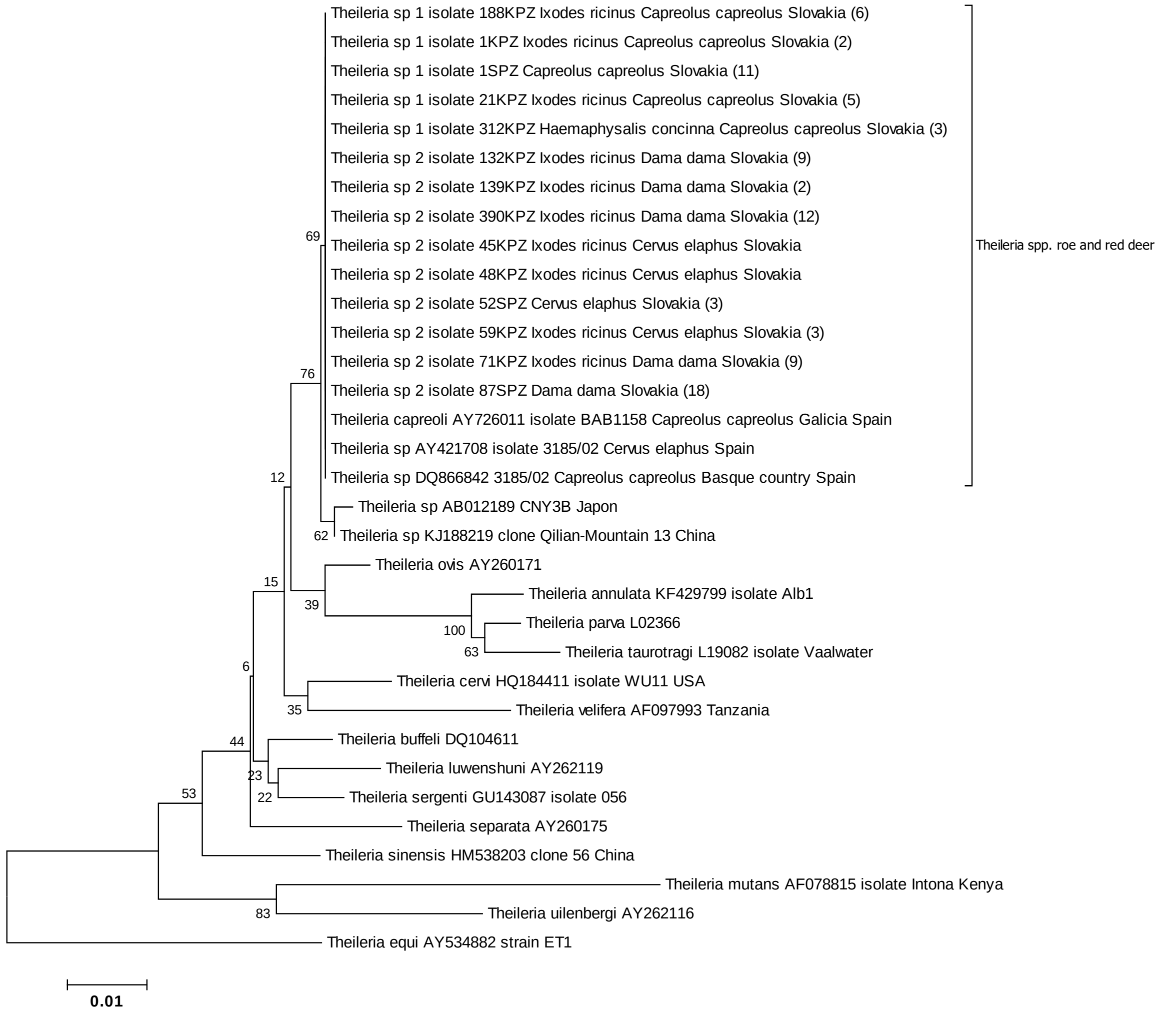

Figure S3 Neighbour-joining tree of hypervariable 18S rRNA gene sequences of *Theileria* parasites using neighbour-joining.

The sequences of isolates from Slovakia are labelled with isolate designation, tick and/or vertebrate host, geographic origin, and number of identical sequences (in brackets). The bootstrap values based of 1,000 replicates are displayed next to the branches. The tree is rooted using *Theileria equi* as the outgroup. Clades displaying a bootstrap value of ≥ 75 are considered relevant. The evolutionary distance is shown in the units of the number of base substitutions per site.
